# Transcriptome Analysis of *Onobrychis viciifolia* During Seed Germination Reveals GA_3_-Inducible Genes Associated with Phenylpropanoid and Hormone Pathways

**DOI:** 10.3390/ijms26052335

**Published:** 2025-03-06

**Authors:** Yanyan Luo, Kun Wang, Jiao Cheng, Lili Nan

**Affiliations:** Pratacultural College, Gansu Agricultural University, 1 Yinmen Village, Anning District, Lanzhou 730070, China; 1073323010016@st.gsau.edu.cn (Y.L.); wangkun@st.gsau.edu.cn (K.W.); chenjiao12389@163.com (J.C.)

**Keywords:** *Onobrychis viciifolia*, seed development, exogenous gibberellin, hormone, differentially expressed genes

## Abstract

Sainfoin (*Onobrychis viciifolia*) is a type of leguminous plant with high feeding value. It contains a high concentration of tannins at all growth stages, which can precipitate soluble proteins and form a large number of persistent foams in the rumen, so that ruminant livestock will not develop dilatation disease during green feeding and grazing. The germination rate of *O. viciifolia* seeds is very low under natural conditions. The preliminary experiment showed that 600 mg/L GA_3_ treatment significantly improved the germination rate and seed vitality of sainfoin seeds. In comparison to CK, GA_3_ significantly decreased the relative content of endogenous inhibitors, with the most notable reduction observed in 4-nitroso-N-phenyl-benzenamine. Therefore, we selected the dry seed stage (GZ), imbibition stage (XZ), split stage (LK), and radicle emergence stage (MF) of four different germination stages treated with GA_3_ for transcriptome analysis. RNA-seq identified 1392, 2534 and 4284 differentially expressed genes (DEGs) in GZ vs. XZ, XZ vs. LK, and LK vs. MF, respectively. During seed germination, DEGs are mainly enriched in hormone signaling and phenylalanine biosynthesis pathways, and up-down-regulation of these DEGs may alter hormone and secondary metabolite levels to promote germination. The results of weighted gene co-expression network construction (WGCNA) also indicate that plant hormone signal transduction and phenylpropanoid biosynthesis play a dominant role in GA_3_-induced seed germination. In conclusion, the combined analysis of transcriptomic and physiological indicators provided new insights into seed germination and a theoretical basis for further study of candidate genes.

## 1. Introduction

Sainfoin (*Onobrychis viciifolia*) is a perennial legume of the genus, which is known as the ‘Queen of Forages’ [1]. Sainfoin has a long history of cultivation and is widely distributed mainly in temperate and subtropical regions of Europe, the Middle East, North America, Russia, and northern and northeastern Africa [2]. Sainfoin is recognized as a superior animal feed resource, characterized by its high nutritional value, significant yield, excellent palatability, and strong nitrogen fixation potential [3]. Since its seeds, roots, stems, leaves, and flowers contain condensed tannins [4], it reduces bloating and urinary nitrogen emissions in ruminant animals and promotes the absorption of amino acids when fed to ruminants [5,6]. Sainfoin seeds are generally harvested in the form of pods, which are enclosed in a tough pod layer. Because of the difficulty in peeling off the tough seed pods, seeds are usually sown with pods, resulting in poor water permeability and low germination rates [7]. The level of germination directly affects the yield and quality of sainfoin. Therefore, efficient planting of sainfoin is necessary to take advantage of the plant’s resources by improving seed germination. Improving sainfoin seed germination rate has been a matter of concern.

Germination represents the beginning of plant life. It is highly responsive to changing external conditions, so plants have developed special mechanisms for the regulation of their growth and development, ultimately affecting plant yield. Phase I is the rapid water-absorption stage. Phase II is the lag period during the germination stage. Phase III is the accelerated embryo growth period during the germination stage [8]. Seeds are influenced by different phytohormones that regulate physiology and a complex signaling network during germination. Gibberellin (GA) promotes germination by breaking seed dormancy, while abscisic acid (ABA) plays an opposite role to GA. The size of the relative ratio of GA/ABA determines the ability of seeds to germinate, and changes in the external environment can affect the relative ratio of ABA/GA within seeds, thus influencing the optimal time for seed germination. It has been found that ethylene (ETH) can attenuate ABA-induced seed germination through the ABA signaling pathway. The seed germination process is regulated by various complex signaling networks that integrate various environmental signals into phytohormone signaling pathways [9,10].

As a critical plant growth regulator, GA is instrumental in various aspects of plant growth and development, including seed germination, stem and root growth, and fruit ripening [11]. GA is a diterpenoid substance that can promote cell division and elongation, accelerate leaf bud growth, break seed dormancy, increase seed germination rate, and corrode the seed cuticle wax layer to improve seed coat water permeability and air permeability, enhancing the seed respiration rate and thereby promoting seed germination [12]. The regulatory mechanism of GA_3_-induced seed dormancy release has been studied through transcriptomic analysis of *Fraxinus hupehensis* [13], *Phyllostachys edulis* [14] and *Panax notoginseng* [15]. However, due to species specificity, different species have different mechanisms of seed dormancy and germination. Therefore, the regulatory mechanism of GA_3_ in the release of germination in *O. viciifolia* is worthy of further study.

Currently, transcriptome sequencing technology has been used to analyze mRNA transcript levels during seed germination, thus revealing the transcriptional and systemic regulation of the seed germination process. In this study, the RNA-Seq technique was used to analyze the gene expression characteristics of seeds with GA_3_ treatment at different germination periods. We provide a theoretical basis for improving sainfoin seed germination by elucidating important metabolic pathways and candidate genes associated with GA_3_-induced germination release.

## 2. Results

### 2.1. Effect of GA_3_ on Germination Rate and Seed Vigor

**Figure 1:**
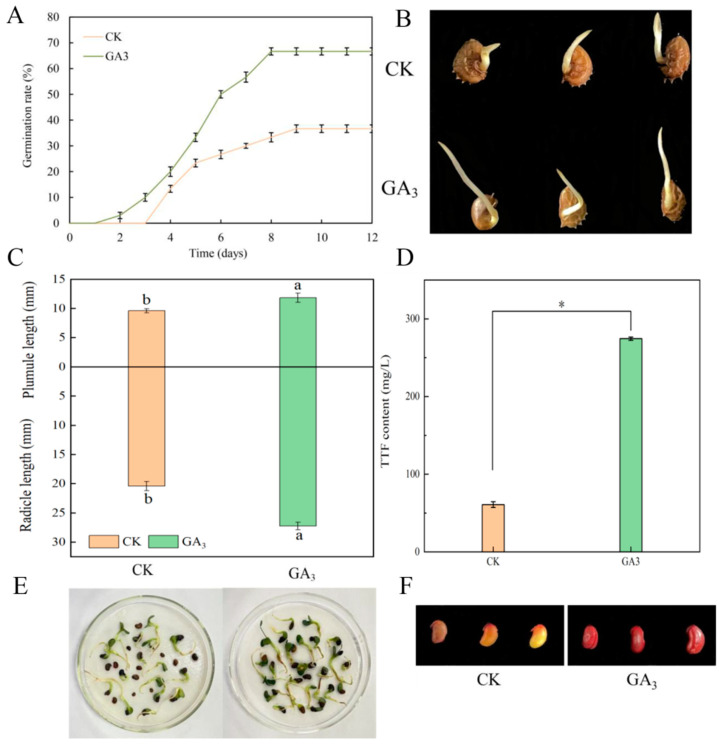
Effects of GA_3_ on the germination rate and seed vigor of sainfoin seeds. (**A**) Effect of GA_3_ treatment on the seed germination phenotype of sainfoin; (**B**) Effect of GA_3_ treatment on seed germination rate; (**C**,**E**) Effect of GA_3_ treatment on plumule and radicle lengths; (**D**,**F**) Effect of GA_3_ treatment on seed viability. Error bars represent the standard deviation (SD) of six replicates. Bars with different lowercase letters were significantly different according to Duncan’s multiple range test (*p* < 0.05). “*” represents a highly significant difference according to the *t*-test.

In this study, seeds were treated with distilled water (CK) or GA_3_ to obtain germinated seeds. The results showed that after 12 days of germination, the germination rates of CK- and GA_3_-treated seeds were 36.67% and 66.67%, respectively (Figure 1A,B). Therefore, GA_3_ treatment significantly improved the germination of sainfoin seeds. There was also a significant increase in plumule length and radicle length (Figure 1C,E), which increased by 23.18% and 33.37% compared to CK, respectively. In order to evaluate the effect of exogenous GA_3_ on seed viability, triphenyl tetrazolium formazan (TTF) staining, and content changes of GA_3_-treated sainfoin seeds were evaluated. The results showed that the TTF content of seeds treated with GA_3_ was significantly higher than in CK seeds (Figure 1D,F). These results indicate that exogenous GA_3_ can significantly improve seed vitality and further enhance germination ability.

### 2.2. Effect of GA_3_ on Endogenous Inhibitors in Sainfoin Seeds

**Figure 2:**
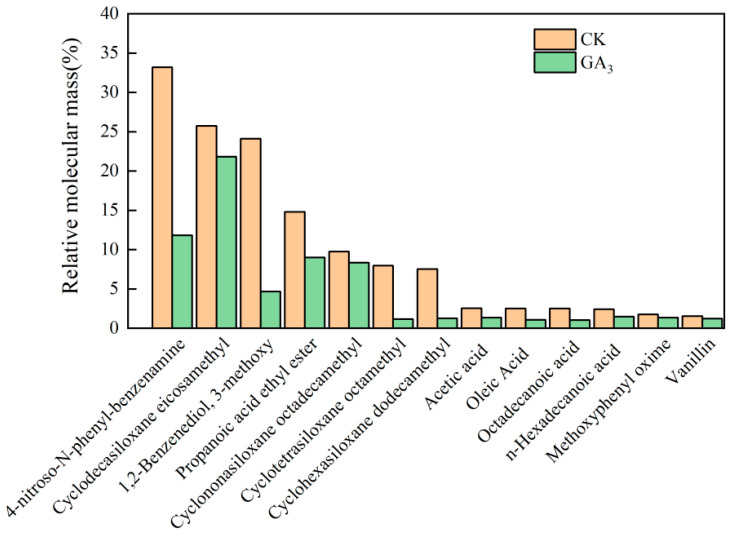
Effects of GA_3_ on endogenous inhibitors in sainfoin seeds.

Through gas chromatography-mass spectrometry (GC-MS) analysis, 13 types of endogenous inhibitors were obtained in the methanol extract of seeds (Figure 2). The highest proportion of 4-nitroso-N-phenylaniline was 33.2% in the CK treatment, and the highest proportion of eicomethyl cyclodecasiloxane was 21.83% in the GA_3_ treatment. Compared to CK, GA_3_ treatment significantly reduced the proportion of endogenous inhibitors in the total mass, and the proportion of 4-nitroso-N-phenyl-benzenamine in the total mass decreased the most—by 180.64%. These results indicated that GA_3_ treatment affected the accumulation of endogenous inhibitors and promoted the germination of sainfoin seeds.

### 2.3. RNA-Sequencing Analysis

To determine the changes in gene expression during GA_3_-promoted seed germination, RNA-Seq libraries were constructed using seed samples at different germination stages (GZ1, GZ2, and GZ3; XZ1, XZ2, and XZ3; LK1, LK2, and LK3; MF1, MF2, and MF3). After sequencing, low-quality readings and joint sequences were removed, resulting in a total of 73.64 GB of clean data. The minimum values of Q20 and Q30 were 99.13% and 97.07%, respectively, and the GC content ranged from 42.45% to 43.98% (Table S2). Thus, the assembly quality of the transcriptome was satisfactory. A total of 83,045 unigenes were obtained after assembly, of which 20,732 unigenes was longer than 1000 bp, and the length of the N50 was 1672 bp (Table S3). By setting the Basic Local Alignment Search Tool (BLAST+2.14.0) parameter E-value to no more than 1 × 10^−5^ and the HMMER parameter E-value to no more than 1 × 10^−10^, a total of 39,022 unigenes with annotation information were ultimately obtained. The number of unigenes annotated in the COG database was 7948 (20.11%), 18,496 (47%) in the KOG database, 37,600 (96.36%) in the NR database, 29,489 (75.57%) in the eggNOG database, 22,253 (57.03%) in the SwissProt database, 23,466 (60.13%) in the KEGG database, 30,497 (78.15%) in the GO database, and 23,397 (59.96%) in the Pfam database. A total of 39,022 unigenes were annotated in public databases, accounting for 47% of the total (83,045 unigenes) (Table S3). The relatively low annotation rate may be due to the specificity of sainfoin and the current limitations in database coverage. These results suggest that the functional annotation of sainfoin genes is still incomplete and that this study plays an important role in filling this gap.

### 2.4. Identification of DEGs at Different Germination Stages

**Figure 3:**
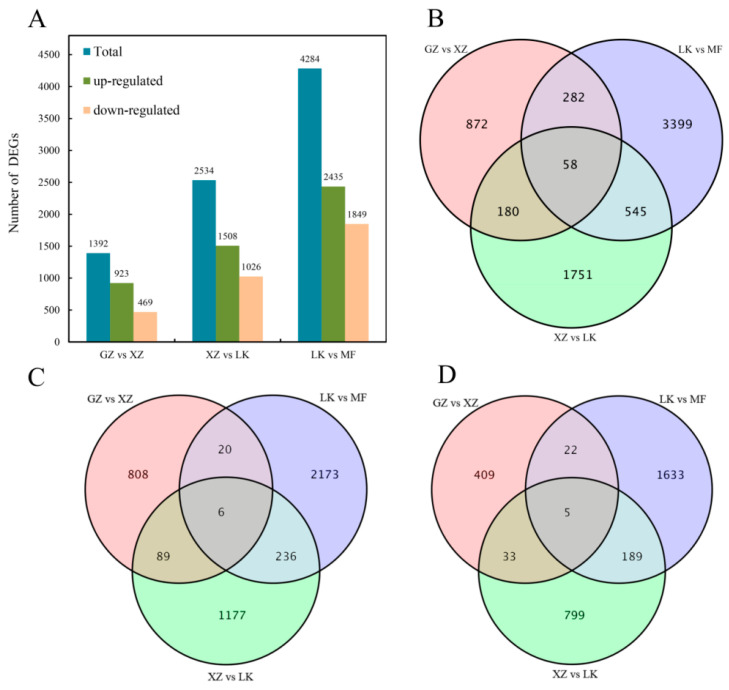
Statistical analysis of differentially expressed genes during germination of sainfoin seeds under GA_3_ treatment. (**A**) Number of total genes, up- and down-regulated DEGs in different comparison groups; (**B**) Venn diagram showing co-expressed genes of the three comparison groups; (**C**) Venn diagram of up-regulated genes; (**D**) Venn diagram of down-regulated genes. DEGs, differentially expressed genes; up, up-regulated DEGs; down, down-regulated DEGs.

After data collation, three comparison groups, including GZ vs. XZ, XZ vs. LK, and LK vs. MF, were used for subsequent analysis. A fold change ≥ 2 and FDR < 0.01 were used as criteria for differential gene screening. There were 1392, 2534, and 4284 DEGs in GZ vs. XZ, XZ vs. LK, and LK vs. MF, respectively (Figure 3A). Of these DEGs, there were 923, 1508, and 2435 up-regulated genes and 469, 1026, and 1849 down-regulated genes. The Venn diagram shows unique and common DEGs among the different comparison groups. A total of fifty-eight DEGs were co-expressed among the three comparison groups (Figure 3B), with six up-regulated genes and five down-regulated genes co-expressed in the three comparison groups (Figure 3C,D). These results suggest that these co-expressed DEGs may play an important role in GA_3_-induced dormancy release.

### 2.5. GO and KEGG Enrichment Analysis

**Figure 4:**
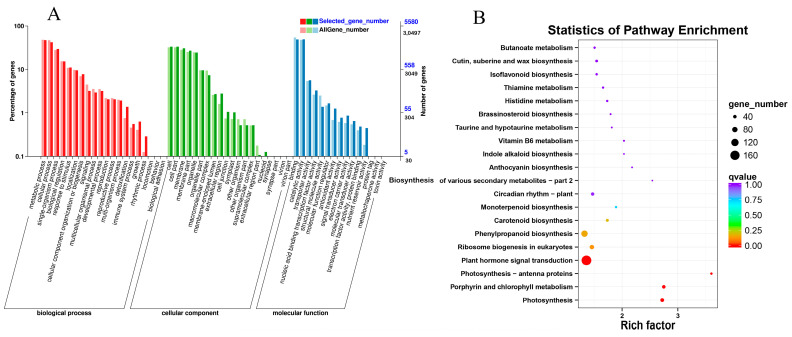
GO and KEGG enrichment analysis of DEGs during germination. (**A**) GO analysis of DEGs in GA_3_-treated sainfoin seeds during the germination process; (**B**) KEGG analysis of DEGs in GA_3_-treated sainfoin seeds during germination process.

To obtain complete functional information, GO enrichment analysis of DEGs was performed (Figure 4A). GO provides an accurate and comprehensive description of gene product function, which helps to understand the distribution of gene function at a macroscopic level. The results showed that most DEGs were enriched in biological processes, molecular functions, and cellular components. In the classification of biological processes, DEGs involved in metabolism, cellular biological processes, single-organism processes, and bioregulation were more abundant. The number of unigenes involved in cellular, membrane, and organelle parts is relatively high in cellular components. In addition, there are major molecular functions such as binding, catalyzing and transporter activities that play a major role in the seed germination process.

KEGG pathway analyses will help to further understand the biological functions of the genes. In order to functionally classify and assign pathways to genes in GA_3_-treated seeds during germination, KEGG analysis was performed on all DEGs (Figure 4B). By comparing to standard reference pathways, the top four KEGG pathways in terms of the number of all DEGs included plant hormone signal transduction, phenylpropanoid biosynthesis, ribosome biogenesis in eukaryotes, and photosynthesis, with the most enriched in hormone signal transduction and phenylpropanoid biosynthesis pathways. Thus, DEGs in hormone signal transduction and phenylpropanoid biosynthesis are intimately involved in GA_3_-promoted seed development, and genes in these pathways were further investigated.

### 2.6. DEGs Involved in Plant Hormone Signal Transduction

**Figure 5:**
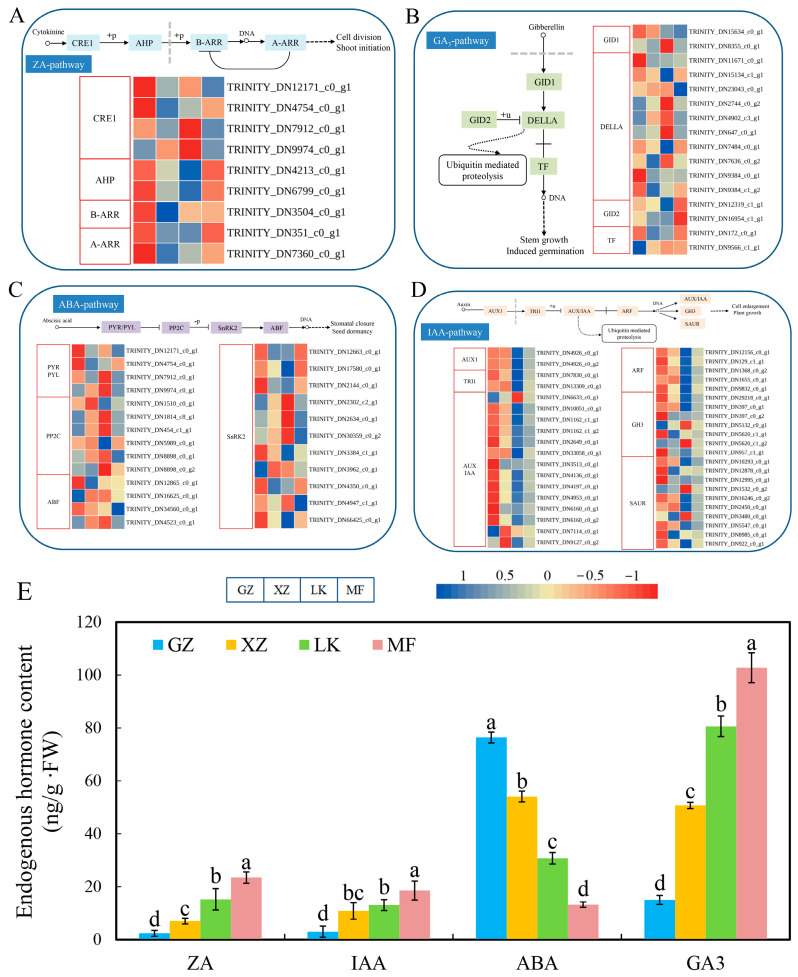
Analysis of hormone signal transduction pathways and endogenous hormone concentrations involved in the germination of sainfoin seeds. (**A**) Expression profile of DEGs associated with the ZA pathway; (**B**) Expression profile of DEGs associated with GA pathway; (**C**) Expression profile of DEGs associated with ABA pathway; (**D**) Expression profile of DEGs associated with IAA pathway; (**E**) Comparison of ZT, IAA, GA_3_, and ABA concentrations in sainfoin seeds under GA_3_ treatment. Sample names are shown at the bottom of the figure. Expression levels, ranging from blue to red, indicate high to low expression of genes. Different lowercase letters indicate significant differences between GA_3_-treated seeds at different germination stages according to Duncan’s multiple range test (*p* < 0.05). The error bar represents three repeated standard deviations (SD).

KEGG annotation results indicate that DEGs in plant hormone signal transduction correlate significantly with GA_3_-induced seed germination. In order to identify DEGs that are correlated significantly in response to GA_3_ treatment, this study analyzed DEGs associated with auxin (IAA), abscisic acid (ABA), zeatin (ZA), and gibberellin (GA) signal transduction. In the ABA signaling pathway, the abscisic acid receptor (PYR/PYL) and protein phosphatase 2C (PP2C), abscisic acid-responsive transcription factor (ABF), and sucrose non-fermenting-1-related protein kinase 2 (SnRK2) play major roles (Figure 5A). Compared with the GZ stage, *OvPP2C* showed a downward trend, and *OvPYR/PYL* genes included both down-regulated genes (TRINITY_DN7912_c0_g1, Trinity_DN7912_c0_g1, TRINITY_DN9974_c0_g) and up-regulated genes (TRINITY_DN12171_c0_g1 and TRINITY_DN4754_c0_g1). However, *OvSnRK2* showed a different expression trend at different stages of germination, and *OvABF* genes were highly expressed in the GZ stage. In the IAA signaling pathway, ten auxin response proteins (SAURs), seven indole-3-acetate amide synthetase (GH3), five auxin response factors (ARFs), and fourteen early auxin-responsive genes (AUXs/IAAs) showed an increasing trend during seed germination (Figure 5B). In the GA biosynthesis pathway, the gibberellin receptor (*GID1*) was highly expressed in the MF stage, and *OvDELLA* was down-regulated during germination (Figure 5C). Therefore, the expression levels of *OvDELLA* and *OvGID1* changed to increase the germination of sainfoin seeds. In the ZA signaling pathway (Figure 5D), cytokinin receptor (CRE1) is highly expressed in the XZ and MF stage, phosphotransporter protein (AHP) is highly expressed in the LK stage, and type-A response regulators (A-ARRs) and type-A response regulators (B-ARRs) are highly expressed in the LK stage, which is significantly higher than that in the GZ stage. These results indicate that the identified DEGs accelerated the germination of sainfoin seeds, and GA_3_ treatment can promote the biosynthesis of ZT by regulating key genes.

In order to investigate the mechanism of action of endogenous hormones in seed germination, we tested the contents of ZT, IAA, GA_3_, and ABA in GA_3_-treated sainfoin at different germination stages (Figure 5E). The results suggested that the contents of ZA, GA_3_, and IAA increased significantly during germination, while the contents of ABA decreased significantly during germination. The GA_3_ content was 1.27, 2.02, and 6.83 times higher in the MF stage than in the LK, XZ, and GZ stages. In comparison with the GZ stage, ABA content declined by 29.18% in the XZ stage, 59.72% in the LK stage, and 82.64% in the MF stage. Exogenous GA_3_ induced seed germination by increasing IAA, ZT, and GA_3_ concentrations and decreasing ABA concentrations. Hence, the changing trend of GA_3_, IAA, ABA, and ZA contents was consistent with the transcriptome data.

### 2.7. DEGs Involved in Phenylalanine Biosynthesis Metabolism

**Figure 6:**
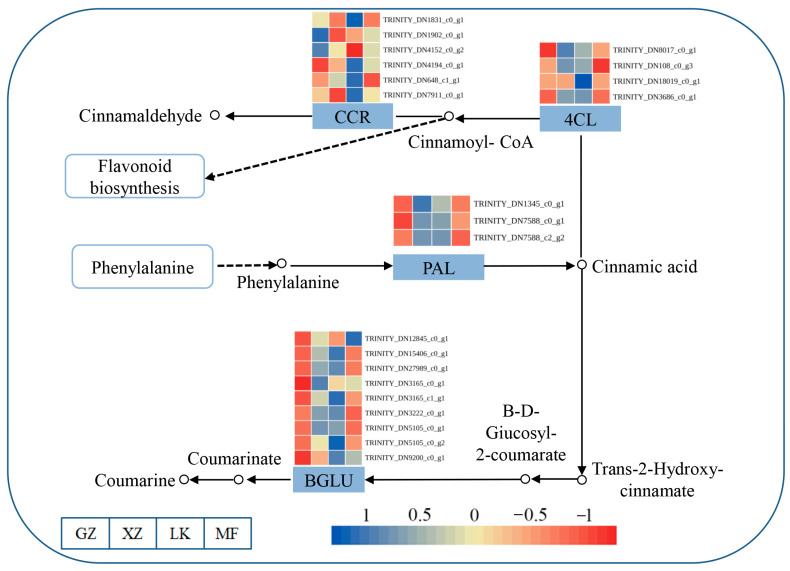
Analysis of phenylalanine biosynthesis pathways involved in the germination of sainfoin seeds. Sample names are shown at the bottom of the figure. Expression levels, ranging from blue to red, indicate high to low expression of genes. All data shown indicate the average mean of three biological replicates (*n* = 3).

In this study, DEGs associated with the phenylalanine biosynthesis pathway were examined to determine their function during GA_3_-promoted seed germination (Figure 6). The DEGs enriched in the phenylalanine biosynthesis pathway were cinnamoyl coenzyme a reductase (*CCR*), 4-coumarate-Co A ligase (*4CL*), phenylalanine ammonia-lyase (*PAL*), and beta-glucosidase (*BGLU*). These genes were highly expressed in the XZ and LK stages of seed germination, especially the expression levels of *OvCCR* genes (TRINITY_DN4194_c0_g1, TRINITY_DN648_c1_g1, TRINITY_DN7911_c0_g1), which were significantly increased in the LK stage. TRINITY_DN3165_c1_g1, TRINITY_DN5105_c0_g1, and TRINITY_DN9200_c0_g1 of the *OvBGLU* gene were not expressed during the GZ period. These results indicate that phenylalanine biosynthesis occurs in the XZ and LK stages, which regulate GA_3_-induced seed germination.

### 2.8. Weighted Gene Co-Expression Network Construction (WGCNA)

**Figure 7:**
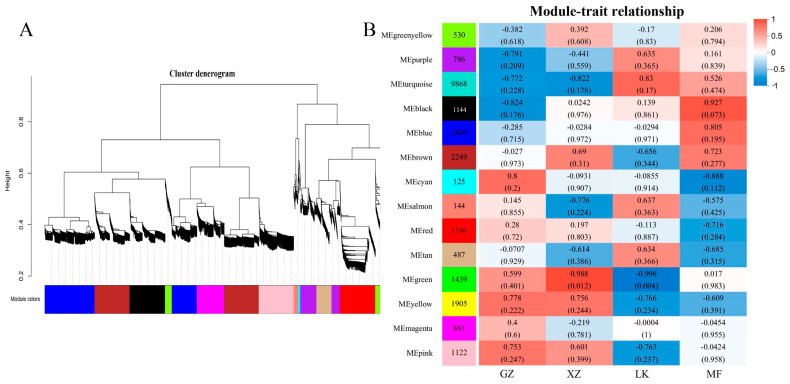
WGCNA of genes during GA_3_-treated sainfoin seed germination. (**A**) Cluster dendrograms showing the co-expression modules identified by WGCNA. Each leaf in the tree represents a gene. Branches correspond to highly interconnected gene modules. The color rows below the dendrograms represent the division of modules based on clustering results and the 14 merged modules based on hierarchical clustering; (**B**) Module-sample relationship based on the Pearson correlation coefficient. Each row corresponds to a module and is represented by a different color. Each column corresponds to samples from different stages of seed germination.

In order to screen the genes associated with GA_3_ treatment during seed germination, WGCNA was used to analyze the related modules. Based on the co-expression patterns of genes, a cluster dendrogram was constructed, and 14 distinct modules were identified, with genes within the same module exhibiting high correlation coefficients (Figure 7A). Next, based on molecular eigenvalues, the expression patterns of each module were analyzed in different samples (Figure 7B). Four modules showed high correlations: MEblack for the MF stage (r = 0.927), MEturquoise for the LK stage (r = 0.83), MEgreen for the XZ stage (r = 0.988), and MEcyan for the GZ stage (r = 0.8). It was shown that the genes in these modules regulated the seed germination of GA_3_-treated seeds. KEGG enrichment revealed that many genes in the MEblack, MEturquoise, MEgreen, and MEcyan were clustered into plant hormone signal transduction, phenylpropanoid biosynthesis, MAPK signaling pathway, plant-pathogen interactions, and starch and sucrose metabolism (Table S4). These results indicate that these key genes regulating plant hormone signal transduction and phenylpropanoid biosynthesis are involved in GA_3_-induced sainfoin seed germination.

### 2.9. Analysis of RNA-seq Data Reliability Using qRT-PCR

**Figure 8:**
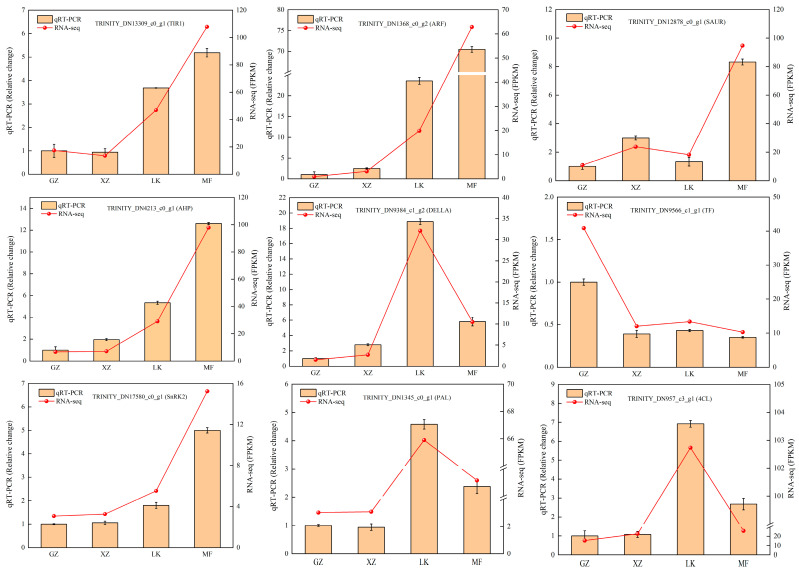
qRT-PCR validation of nine candidate DEGs. The error bar represents three repeated standard deviations (SD).

To verify the accuracy of the DEGs screened from the transcriptome data, we randomly selected nine DEGs related to plant hormone signaling and phenylalanine biosynthesis for qRT-PCR analysis. Specific primers were designed using Primer 5.0 software (Supplemental Table S1). qRT-PCR results showed that the expression patterns of the nine genes were consistent with the RNA-seq data (Figure 8), and only the absolute multiplicity of gene expression was different, confirming the high accuracy of the data obtained from the transcriptome analysis.

## 3. Discussion

### 3.1. GA_3_ Promotes Germination and Attenuates the Accumulation of Endogenous Inhibitors

It is a known fact that seed germination is controlled by phytohormones [16,17,18]. Plant hormones affect seed germination, and exogenous application of GA_3_ can significantly promote the germination of wild *Pistacia vera* and *Fraxinus hupehensis* seeds [19,20]. This study is consistent with the results of previous studies. Compared with CK, 600 mg/L GA_3_ increased the germination rate, germ and radicle lengths of sainfoin seeds. It was also found that 200 mg/L GA_3_ effectively improved the germination rate of *Acer mono* seeds [21]. 150 mg/L GA_3_ resulted in the highest germination rate, germination index, and vitality index of *Nitraria tangutorum* seeds [22]. In our study, we found that 600 mg/L GA_3_ improved the viability of sainfoin seeds, which was also confirmed by Wang et al. [23]. These results indicate that exogenous GA_3_ can significantly enhance seed vitality and further improve germination ability.

We also found that GA_3_ can reduce the variety and relative content of organic compounds in the seeds. These organic compounds can delay or inhibit the germination of seeds and are called endogenous inhibitors. They are produced by the plant itself or other plants in the environment in which the plant lives [24]. Endogenous inhibitors in plants are classified into organic acids, esters, ketones, phenols, quinones, ethers, olefins, volatile aromatic vegetable oils, and gaseous substances according to their chemical structures [25]. In this study, 13 endogenous inhibitors with a higher proportion of total mass were found. Compared with CK, GA_3_ treatment significantly reduced the proportion of the 13 endogenous inhibitors in total mass. Among them, 4-nitroso-N-phenyl-benzenamine and 1,2-Benzenediol-3-methoxy decreased the most. 4-Nitroso-n-phenylaniline belongs to the aniline compound group, which has a strong inhibitory effect on the germination of sainfoin seeds. 1,2-Benzenediol-3-methoxy belongs to phenol organic compounds, and the inhibitory effect of phenol inhibitors generally decreases with the increase in the number of OH groups [26]. 1,2-Benzenediol-3-methoxy belongs to dihydroxyl phenols, and the inhibitory effect is in the middle of phenol substances. It has been found that the seed coat of *Cornus officinalis* contains a large number of phenolics, which affect the diffusion of oxygen and inhibit the germination of seeds [27]. This study demonstrated the effect of GA_3_ on endogenous inhibitors. How exogenous GA_3_ affects the reduction of endogenous inhibitors remains unclear, and further studies will be needed. In summary, our results suggest that 600 mg/L GA_3_ treatment can effectively promote seed germination by stimulating seed embryo development and reducing endogenous inhibitor content.

### 3.2. Plant Hormones Regulate Seed Germination Under GA_3_-Treatment

Hormones are the main members of signal regulation in plants. The molecular synthesis, metabolic pathways, and intermolecular interactions of plant hormones, including ABA, ZA, IAA, GA, JA, CTK, SA, and ETH, are critical for seed germination [28]. Studies have shown that IAA is an important signaling molecule that has synergistic or opposite effects with other hormones during plant growth and collaborates with ABA to promote seed dormancy [29,30]. In combination with IAA content determination and transcriptome expression changes in this study, *OvAUX1*, *OvTIR1*, *OvARF*, most *OvAux/IAAs*, and *OvSUAR* were upregulated in the samples at different stages of germination. The upregulated *OvAUX1* gene is necessary for radicle cell tissue establishment. In the presence of Aux signaling molecules, IAA signaling is activated by the linking of Aux to its receptor protein TIR1, inducing ubiquitin-mediated denaturation of Aux/IAA proteins via the 26S proteasome system, releasing *ARF*, and subsequently stimulating downstream genes [31,32]. In addition, most *OvSAUR* genes were highly up-regulated in response to GA_3_-induced seed germination. The functions of *SAURs* mainly focus on the regulation of plant morphogenesis and IAA signaling and play a role in IAA-mediated cell elongation growth [33]. These results suggest that IAA accumulation may play a positive regulatory role in the germination of sainfoin seeds during germination [34].

GA_3_ regulates various growth and developmental processes, including seed germination and seedling development [35,36]. GA_3_ can break the mechanical restraint of the seed coat and promote radicle protrusion during seed germination [37]. In addition, GA_3_ promotes cell division. Some key genes in the GA_3_ signaling pathway determine seed germination [38]. *GID1*, as a receptor for GA_3_, can form a complex with GA_3_, thereby degrading the growth inhibitor DELLA protein in the GA_3_ signaling pathway [39]. This study demonstrated that GA_3_ treatment up-regulated *OvGID1* expression while down-regulating *OvDELLA* expression. Exogenous GA_3_ reduced OvDELLA protein synthesis by binding with *OvGID1* during seed germination, thus promoting seed germination. Overall, GA_3_ treatment significantly affected the positive regulators in the pathway regulating GA_3_ signaling. This change increased GA_3_ content, and biochemical analyses also confirmed the phenomenon, indicating its role in the germination process of sainfoin seeds.

ABA regulates the accumulation of chemicals in seeds and controls dehydration in later seed development [40]. The ABA content in dry seeds was relatively high and decreased rapidly after germination [41,42]. Minimizing endogenous ABA levels is essential for complete seed germination, as ABA prevents endosperm rupture [43,44]. Exogenous GA_3_ promoted seed germination by reducing the inhibitory effect of ABA [45]. Similar results were obtained in this study, where ABA content was highest in the GZ stage and lowest in the MF stage. ABA binds to its receptor *PYR/PYL* to form a complex. The complex inhibits the action of *PP2C* phosphatase to maintain the phosphorylation of *SnRK2*, thereby enabling *SnRK2* to exert kinase activity to activate downstream transcription factors [46,47]. In this study, four *OvPYL/PYR*, as core regulatory proteins, were heteromorphic during seed germination and were up-regulated in the GZ and XZ stages (TRINITY_DN12171_c0_g1 and TRINITY_DN4754_c0_g1, respectively). In the LK and MF phases, TRINITY_DN7912_c0_g1 and TRINITY_DN9974_c0_g1 were down-regulated. It has been reported that the expression of some *OvPYL/PYR1* genes decreased during the germination of *P. euphratica* and *P. pruinose* [48], which is consistent with the results of this study. Some studies have found that *OvPP2C* is negatively correlated with ABA content and regulation. In this study, the expression level of *OvPP2C* was lower in the GZ stage and higher in the LK stage, which was consistent with the above results. *OvSnRK2* and *OvABF*, as positive regulators in ABA signal transduction pathway, are highly expressed in GZ and XZ phases and play an active role in ABA signal transduction. In summary, exogenous GA_3_ can promote seed germination by up-regulating the negative feedback factor in ABA signaling pathway and reducing ABA content.

ZT is the first type of cytokinin isolated and identified during the growth and development of plants. It mainly exists in seeds, has strong metabolism, and exhibits an antagonistic effect on seed germination inhibitors [49]. The ZT content of pea seeds reaches a peak during germination, especially during radicle protrusion [50]. After GA_3_ treatment, ZT content was highest in the MF stage, followed by LK and XZ stage, and the lowest in GZ stage, indicating that the increase in ZT content promoted the protrusion and elongation of the seed radicle. Four *OvCRE1* genes, two *OvAHP*, one *B-ARR,* and two *A-ARR* are involved in the ZT signal transduction pathway. The up-regulation of *OvAHP* and *OvA-ARR* plays an active role in water absorption and defecation. *B-ARR* is down-regulated in the LK and MF phases. This result may be related to ZT’s mobilization of reserve metabolism and promotion of radicle processes [35]. In conclusion, GA_3_ treatment can accelerate the metabolism of ZT, thus promoting the germination of red bean grass seeds.

### 3.3. Phenylpropanoid Biosynthesis Involved in Seed Germination of GA_3_-Treated Seeds

Previous studies have shown that the phenylalanine biosynthetic pathway is involved in the seed germination process through the production of secondary metabolites such as lignin and flavonoids [51]. It reduces the accumulation of reactive oxygen species during seed germination and provides the energy required for seed germination [52]. *PAL* and *4CL* play a dominant role in phenylpropanoid biosynthesis [53]. In this study, KEGG pathway analysis showed that 22 DEGs were enriched in phenylpropanoid biosynthesis pathways, suggesting that phenylpropanoid biosynthesis is a key pathway for GA_3_-induced seed germination. Chen et al. [54] also described similar results and found that the phenylpropanoid biosynthesis pathway plays an important role in *Cunninghamia lanceolat* seed germination. In addition, most DEGs in phenylpropanoid biosynthesis were up-regulated during seed germination. Tong et al. [55] found that inhibition of phenylpropanoid biosynthesis by Bruceine D decreased *Bidens pilosa* seed germination, suggesting that the activity of key enzymes in the phenylpropanoid biosynthesis pathway plays a major role in the germination and early axial growth of seeds.

**Figure 9:**
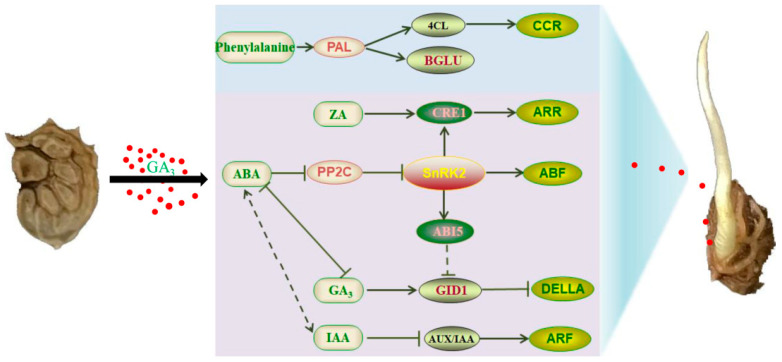
Regulation model of exogenous GA_3_ treatment promoting sainfoin seed germination.

In conclusion, exogenous GA_3_ increased the germination rate and seed vigor, decreased the relative content of endogenous inhibitors, and promoted the germination of sainfoin seeds. The transcriptomic approach was used to study the effect of GA_3_ on sainfoin seed germination, and many DEGs involved in seed germination were obtained by comparing the transcriptomes of the seeds. Among them, the plant hormone signal transduction pathway was one of the main pathways enriched in the KEGG pathway, which has been identified as related to IAA (*OvSAUR*, *OvARF*), ZA (*OvCRE1* and *OvARR*), ABA (*OvPYL*, *OvSnRK2,* and *OvPP2C*), and GA (*OvGID1* and *OvDELLA*) signaling-related genes as the main targets of GA_3_-induced germination. Genes involved in the phenylalanine biosynthesis pathway (*OvPAL*, *Ov4CL*) were activated, contributing to maintaining ROS homeostasis and enhancing resistance during GA_3_-induced germination. In summary, based on transcriptome and physiological index analysis, a regulatory model of GA_3_-induced seed germination of sainfoin was proposed (Figure 9). The application of exogenous GA_3_ can induce the expression of DEG genes and change the GA/ABA ratio, thereby increasing the content of endogenous GA, thus activating the phenylalanine biosynthesis system, maintaining ROS homeostasis and enhancing the activity in vivo. The results of this study will deepen our understanding of the molecular mechanisms of GA_3_-induced seed germination and further enrich our understanding of seed dormancy and germination.

## 4. Materials and Methods

### 4.1. Plant Materials and Treatments

The full and uniformly sized seeds of *O. viciifolia* cv. Gansu were sterilized with a 10% NaClO solution for 10 min and rinsed several times with sterile water. Distilled water (as a control) and 600 mg/L GA_3_ aqueous solution were then added, and the samples were soaked at 4 °C for 24 h with intermittent stirring. The soaked seeds were placed in a Petri dish with a diameter of 9 cm and two layers of filter paper, and 5 mL of distilled water was added. The Petri dishes were placed in a light incubator at a temperature of 25 °C and a relative humidity of 80% for germination tests. To keep the Petri dish moist, the germination bed was sprayed with distilled water daily. The sainfoin seeds with radicles greater than 2 mm were counted to calculate the germination rate. All germination experiments had six biological replicates of 30 seeds each.

Based on the pre-experiment on the seed germination of sainfoin, seed germination of GA_3_-treated seeds was classified into four processes: dry seed stage (GZ), imbibition stage (XZ), split stage (LK), and radicle emergence stage (MF), which corresponded to the days of germination of 0, 2, 6 and 10 days, respectively. Seed samples were collected during these four germination stages. The treated samples were frozen with liquid nitrogen and stored at −80 °C for transcriptome and physiological index analysis. All seed samples consisted of three biological replicates, with each replicate containing 15 g of germinated seeds.

### 4.2. Determination of Seed Vigor, ZT, IAA, GA_3_, and ABA Contents in Seeds

Seed viability was determined using the triphenyltetrazolium chloride (TTC) method. CK- and GA_3_-treated seeds were removed from the pod skin and seed coat to obtain the embryo, soaked in TTC for 2 h, and then photographed, weighed, and ground into powder with liquid nitrogen. The wavelength was measured using a spectrophotometer at 484 nm, and the content of triphenyl tetrazolium formazan (TTF) was calculated [56]. The contents of ZT, IAA, GA_3_, and ABA were measured using high performance liquid chromatography (HPLC) [57].

### 4.3. Extraction and Determination of Endogenous Inhibitors

Ten grams of sainfoin seeds were sterilized with a 10% NaClO solution for 10 min and rinsed several times with sterile water. Then, the seeds were soaked in distilled water (as a control) and in a 600 mg/L GA_3_ solution for 24 h, respectively. The seeds were then crushed in a pre-cooled mortar, and 50 mL of an 80% methanol solution was added and left to stand at 4 °C for 24 h. The supernatant was obtained by centrifugation and then concentrated under reduced pressure at 60 °C. Finally, the concentrated liquid was adjusted to 10 mL with methanol. The concentrated samples were identified by gas chromatography-mass spectrometry (GC-MS).

### 4.4. cDNA Library Construction and Transcriptome Sequencing

Total RNA from GA_3_-treated sainfoin seeds were extracted using an RNA extraction kit (Tiangen, Beijing, China) according to the method provided by the manufacturer. The quantity and integrity of RNA were assayed using a NanoDrop 2000 spectrophotometer (Thermo Fisher Scientific, Waltham, MA, USA) and an Agilent Bioanalyzer 2100 (Thermo Fisher Scientific, MA, USA). Subsequent enrichment of mRNA, dNTP reverse transcription, complementary strand cDNA synthesis, addition of adenine and junction end repair, complementary strand degradation, and fragment enrichment were performed to construct the library. Finally, the library quality was assessed using the Agilent Bioanalyzer 2100 system. The clustering of the index-coded samples was performed on a cBot Cluster Generation System using the TruSeq PE Cluster Kit v3-cBot-HS (Illumia, San Diego, CA, USA) according to the manufacturer’s instructions. After cluster generation, sequencing was performed in PE150 mode for each transcriptome sample, with three replicates using a high-throughput sequencing platform (Illumina NovaSeq 6000), generating paired-end reads. The work was carried out by Biomarker Technologies Co., LTD (Beijing, China).

### 4.5. De Novo Assembly and Functional Annotation of Unigenes

After sequencing, raw data (raw reads) in fastq format were first processed through in-house perl scripts. In this step, clean data (clean reads) were obtained by removing reads containing adapters, reads containing ploy-N, and low-quality reads from the raw data. At the same time, Q20, Q30, GC content, and sequence duplication levels of the clean data were calculated. All downstream analyses were based on clean data of high quality. The left files (read1 files) from all libraries/samples were pooled into one large left.fq file, and the right files (read2 files) into one large right.fq file. Transcriptome assembly was accomplished based on the left.fq and right.fq files using Trinity (2.14.0) with min_kmer_cov set to 2 by default and all other parameters set to default (—min_contig_length 200, —group_pairs_distance 500). The 83,045 unigenes were aligned against databases such as NR (NCBI non-redundant protein sequences), Pfam (Protein family), KOG/COG/eggNOG (Clusters of Orthologous Groups of proteins), Swiss-Prot (A manually annotated and reviewed protein sequence database), KEGG (Kyoto Encyclopedia of Genes and Genomes), and GO (Gene Ontology) using DIAMOND to obtain the functional annotations of unigenes.

### 4.6. Determination of Differential Expression Genes

After completing the assembly, we used Bowtie [58] to align sequencing reads to the unigene library. Based on the alignment results, we calculated the FPKM (Fragments Per Kilobase of transcript per Million mapped reads) values using RSEM [59]. FPKM [60] represents the number of reads per kilobase of transcript length per million mapped reads, which is a commonly used method for estimating gene expression levels in transcriptome sequencing data analysis. Subsequently, we performed a differentially expressed genes (DEGs) analysis using the calculated FPKM values. DEGs were calculated using the DESeq R software package (1.10.1). During the detection of DEGs, a fold change ≥ 2 and FDR < 0.01 were used as the screening criteria, and the differentially expressed genes were functionally annotated and analyzed for enrichment.

### 4.7. qRT-PCR Analysis

To verify the accuracy of the transcriptomic data, 9 DEGs involved in the germination of sainfoin seeds were selected for qRT-PCR analysis. The total RNA was reverse-transcribed into cDNA using a cDNA synthesis kit (Tiangen, Beijing, China). We used the LightCycler 480 Real-Time PCR System (Roche Applied Science) (Tiangen, Beijing, China) and the SYBR^®^ Green Premix Pro Taq HS qPCR Kit (Tiangen, Beijing, China) for the qRT-PCR experiment [61]. There were three biological replicates per gene. The primers were designed using Primer 5, and their specificities were confirmed by a BLAST search. As shown in Table S1, the sainfoin *OvActin* gene was used to normalize relative expression levels. The relative expression of each gene was analyzed using the 2^−∆∆CT^ method [62].

### 4.8. Weighted Gene Co-Expression Network Analysis (WGCNA)

After filtering out undetectable or low-expression genes, the “WGCNA” R package was used to perform WGCNA analysis on genes with FPKM values greater than 0.5 [63]. Soft thresholds were selected based on scale independence above 0.8. A hierarchical clustering tree was constructed using the dissimilarity matrix between genes, and then they were assigned to different modules by dynamic clipping with default parameters set to minimum module size = 30 and clipping height = 0.25.

### 4.9. Statistical Analysis

SPSS 22.0 software was used to perform a one-way analysis of variance (ANOVA) on the experimental data. Excel 2010 was used to analyze and calculate the data. The Duncan method was used to analyze the difference in significance (*p* < 0.05).

## Data Availability

The Illumina sequencing data used in this study have been submitted to the BioProject database of the National Center for Biotechnology Information (PRJNA1205502, https://www.ncbi.nlm.nih.gov/sra/PRJNA1205502, accessed on 2 February 2026).

## Supplementary Materials

The following supporting information can be downloaded at: https://www.mdpi.com/article/10.3390/ijms26052335/s1.
